# The use of patient-reported outcome measures in recurrent diverticulitis

**DOI:** 10.1016/j.surg.2025.109492

**Published:** 2025-06-16

**Authors:** Megan Shroder, Thomas Ueland, Liping Du, Ashley Spann, Samuel A. Younan, Allison McCoy, Fei Ye, Alexander T. Hawkins

**Affiliations:** a Division of General Surgery, Section of Colon & Rectal Surgery, Vanderbilt University Medical Center, Nashville, TN; b VA Quality Scholars Fellowship, Geriatric Research Education and Clinical Center (GRECC), Tennessee Valley Healthcare System, Nashville, TN; c Department of Biostatistics, Vanderbilt University School of Medicine, Nashville, TN; d Department of Bioinformatics, Vanderbilt University Medical Center, Nashville, TN; e Division of Epidemiology, Department of Medicine, Vanderbilt University School of Medicine, Nashville, TN

## Abstract

**Background:**

National guidelines for elective colectomy for uncomplicated diverticulitis have changed to a patient-centered approach. Patient-reported outcome measures, such as the Diverticulitis Quality of Life Instrument, may be helpful to determine who will benefit from elective colectomy for diverticulitis.

**Methods:**

We performed a prospective observational cohort study to investigate whether greater Diverticulitis Quality of Life Instrument scores (indicating more severe disease burden) would be associated with increased likelihood of electing to undergo colectomy for recurrent diverticulitis. Adult patients ≥18 year old seen in consultation for uncomplicated diverticulitis at a tertiary referral medical center from March 2021 to August 2023 were included for analysis. The primary outcome of interest was the decision to proceed with elective colectomy vs. continued medical management.

**Results:**

Of the 70 patients included, 48 (68.6%) elected for medical management and 22 (31.4%) had elective surgery planned or completed during the study period. The mean overall Diverticulitis Quality of Life Instrument scores were 4.6 (standard deviation, 1.8) for the medical management group and 5.3 (standard deviation, 1.2) for the elective colectomy group. An adjusted multivariable analysis showed an odds ratio of 1.39 (confidence interval, 1.03–1.89, *P* = .04) for electing surgical management with one-unit greater baseline Diverticulitis Quality of Life Instrument overall score and an odds ratio of 1.36 (confidence interval, 1.03–1.78, *P* = .03) for one-unit greater baseline Diverticulitis Quality of Life Instrument score in the subdomain of behavior.

**Conclusion:**

We observed significantly greater baseline overall Diverticulitis Quality of Life Instrument scores and scores in the subdomain of behavior in patients who chose to pursue elective colectomy after consultation for recurrent diverticulitis.

## Introduction

Diverticular disease is a common process, affecting >30% of adults in the United States and 25% of people worldwide.^1–3^ It is estimated that 200,000 people are hospitalized in the United States with diverticulitis each year.^4^ It is costly to the health care system and carries a significant risk of morbidity for the patients who are affected. Although the rate of emergency surgical procedures for diverticulitis is decreasing, the number of elective surgical interventions is increasing.^5^ Traditionally, it was recommended that elective colectomy should be considered after 2 episodes of diverticulitis to avoid catastrophic recurrence, emergent operative interventions, and stoma formation. However, over the last decade, studies have shown that there is little reduction in mortality with prophylactic colectomy for diverticulitis, shifting the focus of medical and surgical treatments instead toward the prevention of morbidity and increasing the patient’s quality of life.^6–8^

The morbidity and mortality associated with elective colonic resection for diverticular disease previously has been shown to be low, making the standard outcome measures of 30-day survival, wound complications, and in-hospital complications poor metrics to analyze when elective surgical intervention is indicated. Although national recommendations state colectomy should be considered after diverticulitis complicated by fistula, obstruction, stricture, or large abscesses (>5 cm), elective resection is no longer recommended on the basis of the number of episodes of diverticulitis. The American Society of Colon and Rectal Surgeons clinical practice guidelines for management of diverticulitis reflect this in their recommendation for “individualized” decision-making regarding elective colectomy after uncomplicated diverticulitis.^9^ American Society of Colon and Rectal Surgeons recommends considering the severity of episodes, persistent symptoms, operative morbidity, and patient preferences. These recommendations do not include objective measurements to assist patients with determining the appropriate time for elective colectomy. Previous work from our group used Medicare data to evaluate national trends in elective colectomy for diverticulitis and showed a 3-fold variation in standardized colon resection ratios.^10^ This was thought to reflect the lack of strong national guidelines for treatment options in uncomplicated diverticulitis and identified a deficiency in tools to assist with shared decision-making process for surgical treatment of diverticulitis.

Since the Institute of Medicine directed health care to focus on “patient-centered” care in 2001, there has been an increasing focus on using patient-reported outcome measures (PROMs) within the medical field. The most prevalent and well-studied PROM focused on the symptom burden of diverticulitis is the Diverticulitis Quality of Life Instrument (DV-QOL) (Supplementary Appendix 1).^11^ No previous studies have been completed to see whether certain DV-QOL scores are associated with pursuing elective colectomy.

The most appropriate time to surgically intervene for uncomplicated diverticulitis is not clear. Although there have been an increasing number of studies focusing on outcomes in diverticulitis, PROMs have not been studied extensively in the pre- and postoperative setting to describe when surgical interventions will positive impact quality of life. This poses a significant challenge for patients and their providers when predicting when surgery will provide benefit for an individual patient. There is a need for meaningful information regarding quality of life in uncomplicated diverticulitis to aide patients in decision making regarding elective surgical resection. With additional study of the DV-QOL instrument, we believe it can serve as a useful objective data source when patients and surgeons are considering elective colectomy. We hypothesized that greater DV-QOL scores (indicating more severe disease burden) would be associated with increased likelihood of electing to undergo colectomy for recurrent diverticulitis.

## Methods

### Study design and setting

This was a review of data from a single-site tertiary care center. PROMs (DV QOL) were collected prospectively for all adult patients at the Vanderbilt University Medical Center Colorectal Surgery Clinic from March 1, 2021, to August 31, 2023, and other covariates were collected retrospectively for this time period. All study patients completed a DV-QOL survey associated with their clinic visit.

Data were extracted from Vanderbilt University Medical Center’s EPIC electronic health system. This project was reviewed and approved by the Vanderbilt University Medical Center’s Institutional Review Board (study number 230785) with a waiver of informed consent.

### Study Population

Our study population included adult patients (≥18 years old at the time of initial clinic appointment) who were seen in consultation for a diagnosis of acute uncomplicated diverticulitis (*International Classification of Diseases*, *Tenth Revision*, diagnosis code K57.0–9). Exclusion criteria included diagnoses of active malignancy, inflammatory bowel disease, irritable bowel syndrome, history of solid-organ transplant, or complicated diverticulitis. defined in the study as diverticulitis with active colonic fistula, obstruction, stricture, or large abscess (>5 cm).

### Outcome and variables of interest

The primary outcome was decision to proceed with elective colectomy compared with continued observation. Decision to proceed with elective colectomy was defined within the study as completed or scheduled elective colectomy during the study period. Patients who had previously scheduled and subsequently canceled surgeries were included in the medical management group reflective of their ultimate treatment decision. The decision to undergo surgery in this patient population starts with a discussion between the patient and their surgeon at an outpatient clinic visit. After counseling of the patient at this appointment, a shared decision-making process is used. Ultimately, each patient decides if they will pursue elective colectomy after it is determined if surgery is an appropriate treatment plan per the discretion of their individual surgeon.

The exposure in this study is each patient’s baseline DV-QOL score associated with their consultation clinic visit. This instrument was developed in 2015 by Spiegel et al^11^ at UCLA and Cedars-Sinai, this PROM includes a 17-question survey that asks patients about their symptom burden over the last 2 weeks. It is scored on a 0- to 10-point scale with 0 indicating the lowest burden of disease and 10 representing the highest burden of disease. This PROM has 4 subdomains: physical symptoms, patient concerns, emotions, and patient behavior. Other collected covariates included age, sex, race (White/Caucasian, Black/African American, other), ethnicity (Hispanic/Latino/Spanish or other), body mass index, insurance type (private/commercial, Medicaid, Medicare), smoking status (never, prior, current), and Charlson-Deyo Comorbidity Index (CCM).

### Statistical analysis

Patients’ demographics and characteristics were summarized using descriptive statistics, with means ± standard deviation, medians and quartiles for continuous variables, and frequencies and percentages for categorical variables. The 2 groups (medical management and elective colectomy) were compared using Pearson χ^2^ test and Wilcoxon rank-sum test for categorical and continuous variables, respectively. The DV-QOL scores of patients in the 2 groups were demonstrated using dots and boxplots and compared using Wilcoxon rank sum test.

We conducted multivariable logistic regression analysis to assess the association between the decision regarding elective surgery (yes/no) and DV-QOL score, adjusting for age and comorbidity index as potential confounders. The model was prespecified to avoid overfitting. Separate models were fitted with individual DV-QOL subdomain scores (physical symptoms, patient concerns, emotions, and patient behavior). Adjusted odds ratios and corresponding 95% confidence intervals were reported for all multivariable analyses. All analyses were determined on the basis of complete data (no imputation for missing data) and performed with R, version 4.3.1, including R packages: Hmisc, rms, and ggplot2.

## Results

**Figure 1.**
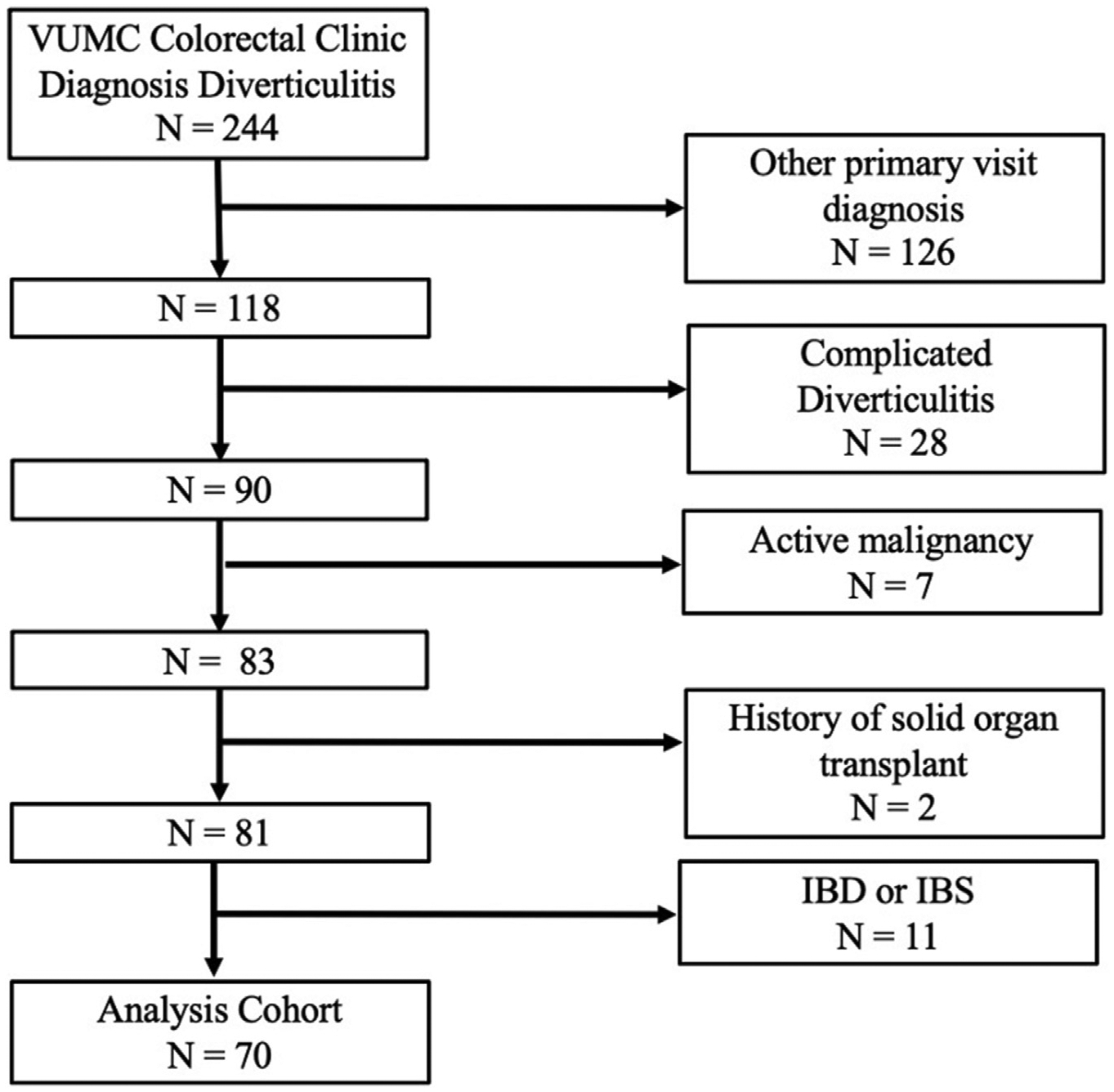
Patient flow diagram. *IBD*, inflammatory bowel disease; *IBS*, irritable bowel syndrome; *VUMC*, Vanderbilt University Medical Center.

There were 244 patients who were seen in the clinic with an active *International Classification of Diseases*, *Tenth Revision*, diagnosis of diverticulitis who completed a DV-QOL survey associated with their clinic appointment. Of these, 70 patients were included in the study on the basis of our inclusion/exclusion criteria (Figure 1). In total, 48 (68.6%) elected for medical management without surgical resection and 22 (31.4%) had elective colectomy planned or completed during the study period (19 completed resections, 3 scheduled for future dates outside of our study period).

**Figure 2.**
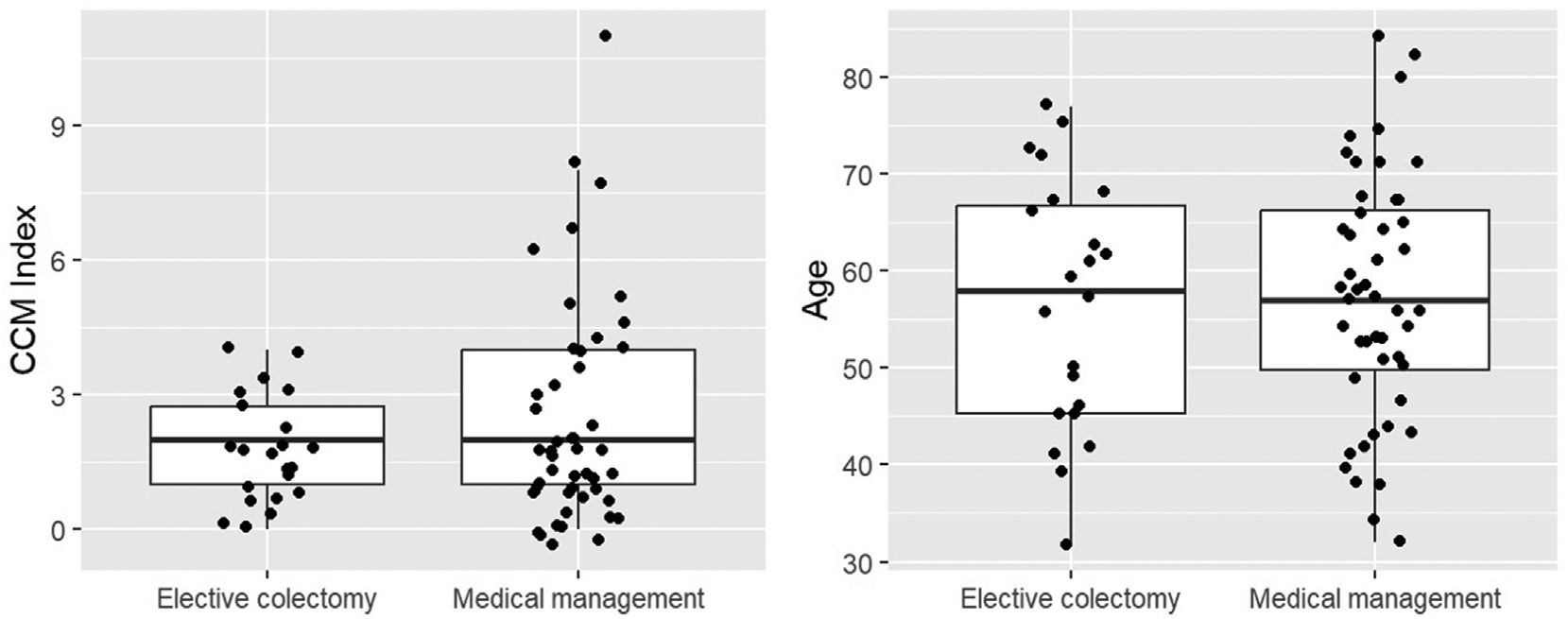
Boxplot of CCM and age by treatment groups. Individual patient data points were overlaid with a boxplot for each group. The middle line in the boxplots stands for the median, the box demonstrates the lower and upper quartiles, and the whiskers expand to the 5th and 95th percentiles. *CCM*, Charlson-Deyo Comorbidity.

The median age of the study cohort was 57 years old (interquartile range [IQR], 46.9–67.0) as demonstrated in Table I. The self-identified sex was female in 42 (60%) of the patients and male in 28 (40%) of the patients. Smoking status was available for 55 of the patients with 56.4% reporting never smoking nicotine-containing products, 38.2% reporting previous use, and 3.6% reporting current use. Charlson Comorbidity Index did not differ between the 2 groups with a median score of 2 for each group, though the IQR was much lower in the surgical group: 0–4 vs 0–11 in the group who elected for continued medical management as seen in Figure 2.

DV-QOL scores were collected for each subject’s initial consultation clinic visit. Of the 70 included patients, 38 (54.2%) completed their DV-QOL on their clinic date and 29 (41.4%) completed it within 3 days of their clinic visit. There were 3 patients (4.3%) who had a longer time period between their clinic visit and DV-QOL completion date (32, 41, and 53 days). Sensitivity analysis was completed excluding these patients from statistical analysis and results were not significantly changed, so these patients were included in the final analysis.

**Figure 3.**
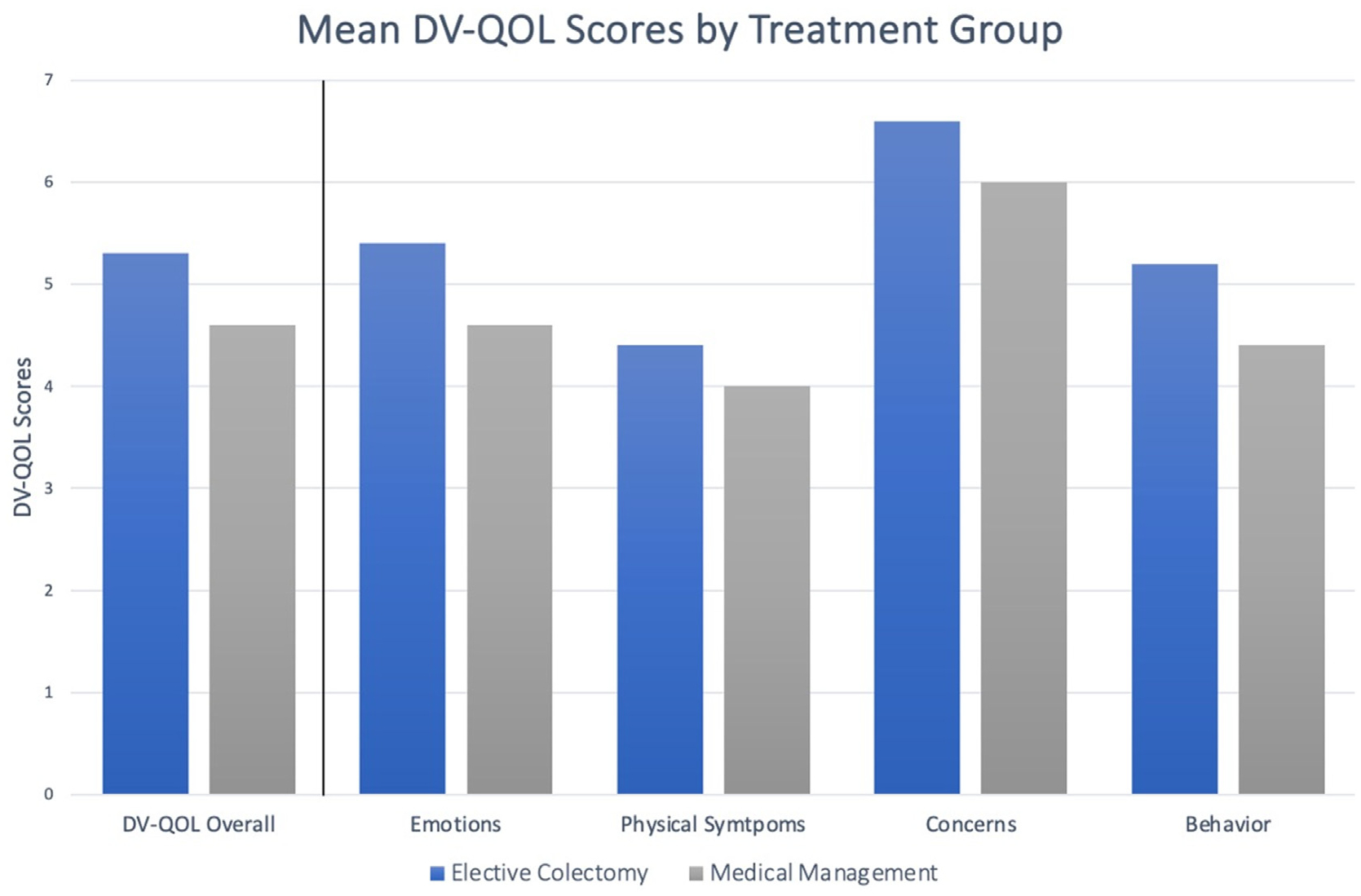
Mean DV-QOL scores for subjects by treatment group. *DV-QOL*, Diverticulitis Quality of Life Instrument.

**Figure 4.**
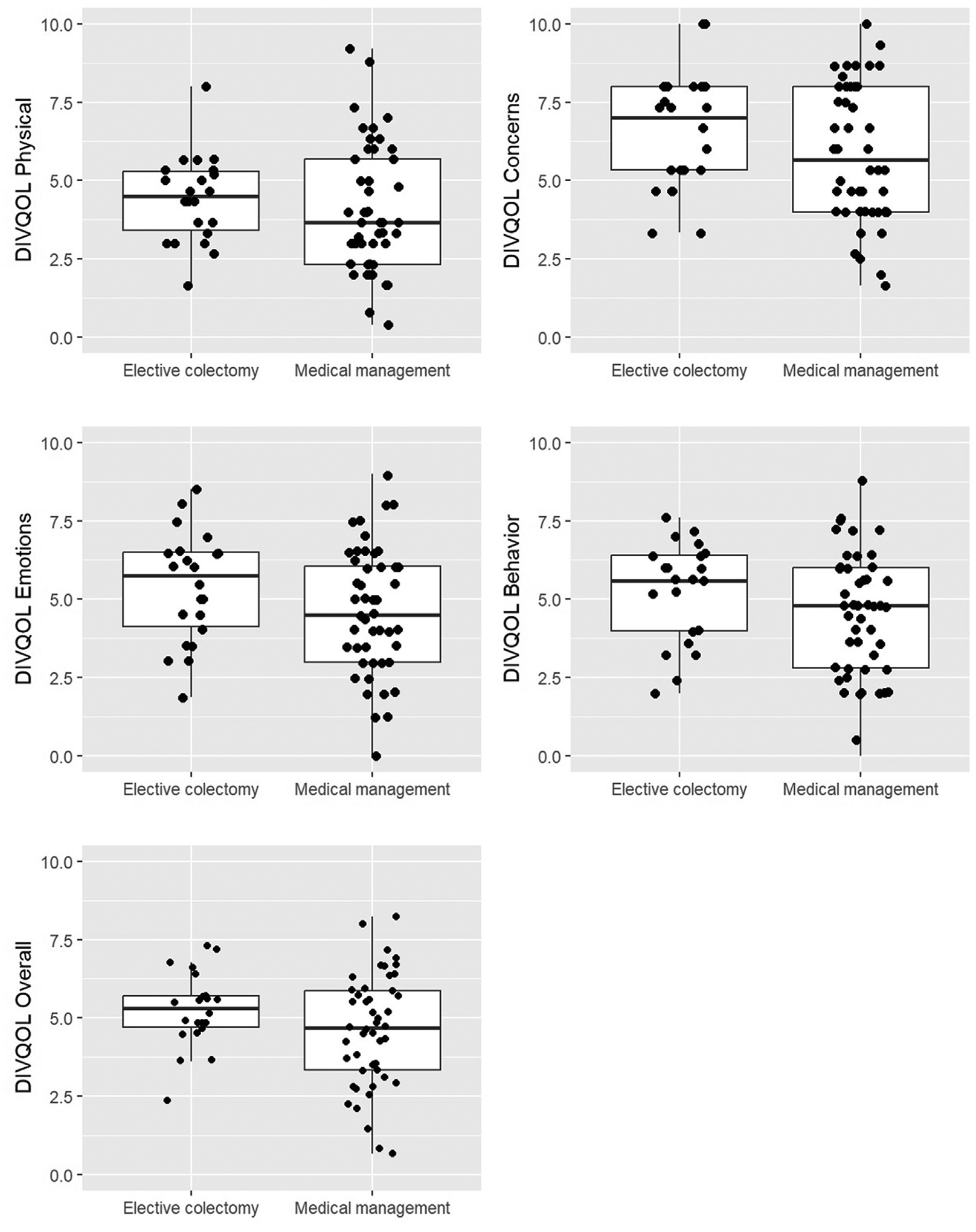
Boxplot of DV-QOL scores treatment group. Individual patient data points were overlaid with a boxplot for each group. The middle line in the boxplots stands for the median, the box demonstrates the lower and upper quartiles, and the whiskers expand to the 5th and 95th percentiles. *DV-QOL*, Diverticulitis Quality of Life Instrument.

The median overall DV-QOL scores were 4.7 (IQR, 3.3–5.9) for those who elected for nonoperative management during the study period and 5.3 (IQR, 4.7–5.8) for those who elected to undergo elective colonic resection as demonstrated in Table II and Figures 3 and 4.

In an adjusted analysis, greater DV-QOL scores were significantly associated with a greater odds of electing surgical management, with an adjusted OR of 1.39 (CI, 1.02–1.89, *P* = .036) for 1-point increase in baseline DV-QOL overall score and an OR of 1.36 (CI, 1.03–1.78, *P* = .028) for 1-point increase in the DV-QOL subdomain behavior score. Covariate adjustment included age and CCM, which were selected a priori on the basis of their clinical relevance as baseline characteristics. The adjusted analyses for other DV-QOL subcategories (physical symptoms, concerns, and emotions) did not show statistically significant associations with electing surgical management.

## Discussion

In this study, we evaluated patients in our tertiary care center who were seen in the Colorectal Surgery clinic in consultation for acute uncomplicated diverticulitis. Using baseline DV-QOL responses and evaluation of each patient’s decision to proceed with either continued medical management or elective colectomy, we observed significantly greater baseline overall DV-QOL scores and greater scores in the subdomain of behavior in patients who chose to pursue elective colectomy after consultation for recurrent diverticulitis. The odds of electing surgical intervention over continued medical management were approximately 40% greater with a 1-point increase in overall DV-QOL scores and behavior subdomain scores. The behavior subdomain of the DV-QOL score includes wearing different clothing, missing work, avoiding social engagements, and losing sleep.

Notably, there were no significant differences in baseline scores in the subdomains of physical symptoms, which covers items such as bloating, diarrhea, nausea, and abdominal pain. These physical symptoms of diverticulitis often are focused on in history gathering but may be less important to decision-making than the items included in the behavior subdomain described previously. In addition, the subdomains of concerns and emotions cover items such as anxiety regarding future flares, frustration, and fears about health consequences. Traditionally, discussion regarding elective surgical management has focused on the fear of future episodes or the need for stoma formation; however, our findings indicate these may not be the correct factors to focus on when counseling patients.

Our findings indicate the DV-QOL instrument, especially the subdomain of behavior, can be a useful tool to gauge the impact of diverticulitis on quality of life and that providers should focus on eliciting information regarding the impact of diverticulitis on a patient’s behavior when counseling them about their treatment options. This could include more targeted questions regarding their disease and its impact on their work and social schedule, daily routine, diet, clothing, and sleep quality and quantity. The impact of this disease process on missing work may also correlate with lost wages and cost of health care episodes for patients with multiple admissions or health care encounters for diverticulitis.

Our findings suggest that although the behavior-related components of a patient’s history with diverticulitis have been previously less emphasized than details about physical symptom severity, these topics may be more influential to patient decision-making. Even in settings without the ability to use or interpret PROMs, such as the DV-QOL instrument, clinicians can use these findings to better focus their history-taking and counseling of patients.

Most patients in our study were examined in clinic several weeks after an acute episode of diverticulitis, so there could be added benefit to collecting DV-QOL instrument data in the future on patients who are actively being treated for acute diverticulitis in the emergency room, urgent cares, primary care offices, or inpatient settings. However, because diverticulitis is treated in many different settings depending on the severity of the episode, coordination of this amount of data collection is difficult and can be costly. The data presented in this study demonstrate that most patients in both groups have continued symptoms/impact of their diverticulitis beyond their acute episode. Although physical symptoms may partially resolve, the psychological and behavioral issues persist. These continued impacts of diverticulitis are shown in this study to impact their decision-making regarding elective surgery.

Previous research from our group has assessed the decision-making process in diverticulitis treatment at our tertiary care center with a qualitative investigation to build a conceptual model.^12^ This study used semistructured interviews of 25 surgeons and multiple patient focus groups focusing on analyzing factors considered by both surgeons and patients when determining if they should proceed with elective colectomy compared with continued medical management. Themes that arose included difficulty in communication, limited knowledge of treatment options, and uncertainty regarding important outcomes. Surgeons emphasized the need for additional tools to assist in decision making as the surgical community evolves from basing their recommendation for resection off objective benchmarks, such as number of episodes to subjective factors, such as quality of life. Our current evaluation of the DV-QOL PROM in the clinic setting is the first step in establishing data focused on quality of life that can be used by providers to improve their interactions with patients during discussions of treatment options.

Previous study of the DV-QOL from Khor et al^13^ in 2021 established meaningful benchmarks within this PROM for significant symptom burden. The study evaluated DV-QOL scores in 177 patients and showed a patient acceptable symptoms state of 3.2 of 10 and a minimal clinically important difference of 2.2, corresponding to patient perception of an important change in their score reflecting a significant change in their burden of symptoms. Although these thresholds are helpful to interpret DV-QOL scores, no further study has been completed to see whether certain DV-QOL scores are predictive of pursuing elective colectomy.

Patients in both groups within our study had baseline scores greater than the previously documented patient acceptable symptoms state of 3.2, indicating a significant burden of disease. Although there were no significant differences in the other subdomain scores between groups in our analyses, the distribution of scores across all domains appeared to be greater in the group who elected for surgical management. Although data collection for this study occurred over 2 years, it is a single-site study, and the study population limits the ability to detect small differences between the groups in other domains.

This study is limited as the study population was majority White/Caucasian (91.4%) and was not diverse in ethnicity with only 3.3% of the study population self-identifying as Hispanic, Latino, or Spanish. As this was a single-site study, the patient population was seen by a group of 8 surgeons working within the same medical center. Although the surgeons at our study site have a diverse range of years in practice and previous training, a larger group of providers from multiple institutions in future studies would allow for more generalizable results. In addition, the sample size of 70 patients limits our ability to control for other variables, investigate postoperative secondary outcomes, and detect small differences between the patient groups. We addressed the risk of overfitting by developing a prespecified model and recognized that the inclusion of additional covariates was constrained by the limited sample size, particularly the subset of patients opting for elective colectomy. With a larger group of patients, we may be able to detect differences between the other domains of the DV-QOL and control for other factors beyond age and CCM.

Future research should focus on evaluating the DV-QOL instrument in a larger population of patients to determine scores predictive of elective colectomy versus continued medical management. In addition, evaluation of decision regret in both treatment groups will be crucial in future research. With a larger group of patients in future studies, we also will be able to evaluate postoperative PROMs and other measures to determine whether the decision to pursue elective colectomy resulted in better outcomes in various subtypes of patients. Previous studies have demonstrated rates of up to 17% of patients with significant decision-regret regarding their choice between medical management and elective colectomy for diverticulitis.^14^ These patients cited psychosocial factors and interactions with physicians as the primary driving forces for their treatment decisions. Although prediction of undergoing future elective colectomy may be helpful in the decision-making process for patients and providers, it is different from predicting long-term agreement with one’s own treatment decision.

In conclusion, we observed significantly greater baseline overall DV-QOL scores and scores in the subdomain of behavior in patients who chose to pursue elective colectomy after consultation for recurrent diverticulitis. These findings indicate the DV-QOL, especially the subdomain of behavior, can be a useful tool to gauge the impact of diverticulitis on quality of life and that providers should focus on eliciting information regarding the impact of diverticulitis on a patient’s behavior when counseling them about their treatment options.

## Supplementary Material

1

Supplementary material associated with this article can be found, in the online version, at [https://doi.org/10.1016/j.surg.2025.109492].

## Figures and Tables

**Table I:** Patient baseline demographics

Demographic variable	Elective colectomy (*n* = 22)	Medical management (*n* = 48)	Overall (*N* = 70)	*P* value
Age, yr				
Median (IQR)	58.0 (45.0–67.1)	57.0 (49.4–66.6)	57.0 (46.9–67.0)	.93*
Range	32.0–77.0	32.0–84.0	32.0–84.0	
Sex				
Female	15 (68.2%)	27 (56.2%)	42 (60.0%)	.34^†^
Male	7 (31.8%)	21 (43.8%)	28 (40.0%)	
Race				
White	20 (90.9%)	44 (91.7%)	64 (91.4%)	.73^†^
Black or African American	2 (9.1%)	3 (6.2%)	5 (7.1%)	
Other	0 (0.0%)	1 (2.1%)	1 (1.4%)	
Ethnicity				
None of these	18 (81.8%)	35 (72.9%)	53 (75.7%)	.58^†^
Hispanic, Latino/a, or Spanish origin	0 (0.0%)	2 (4.2%)	2 (2.9%)	
Prefer not to answer	0 (0.0%)	2 (4.2%)	2 (2.9%)	
Missing	4 (18.2%)	9 (2.1%)	13 (18.6%)	
BMI				
Median (IQR)	30.6 (26.0–34.7)	29.1 (24.1–36.3)	29.4 (25.4–36.1)	.49*
Range	20.0–49.2	16.8–61.3	16.8–61.3	
Insurance payor				
Blue Cross	4 (18.2%)	7 (14.6%)	11 (15.7%)	.54^†^
Blue Cross out of state	2 (9.1%)	1 (2.1%)	3 (4.3%)	
Other commercial	8 (36.4%)	21 (43.8%)	29 (41.4%)	
Medicaid	0 (0.0%)	1 (2.1%)	1 (1.4%)	
Medicare	5 (22.7%)	6 (12.5%)	11 (15.7%)	
Medicare advantage	2 (9.1%)	10 (20.8%)	12 (17.1%)	
Other governmental	1 (4.5%)	1 (2.1%)	2 (2.9%)	
Missing	0 (0.0%)	1 (2.1%)	2 (2.9%)	
Encounter smoking status				
Current use	1 (4.5%)	1 (2.1%)	2 (2.9%)	.75^†^
Quit	8 (36.4%)	13 (27.1%)	21 (30.0%)	
Never	9 (40.9%)	22 (45.8%)	31 (44.3%)	
Missing	4 (18.2%)	12 (25.0%)	16 (22.9%)	
Charlson-Deyo Comorbidity (CCM) index				
Median (IQR)	2.0 (1.0–3.0)	2.0 (1.0–4.0)	2.0 (1.0–3.0)	.72*
Range	0.0–4.0	0.0–11.0	0.0–11.0	

N is the number of nonmissing values. Patients in a category were represented as n/total (percentage).

*BMI*, body mass index; *IQR*, interquartile range; *SD*, standard deviation.

* Wilcoxon.

† Pearson.

**Table II:** DV-QOL scores overall and by treatment group

Measurement	*N*	Elective colectomy (*n* = 22)	Medical management (*n* = 48)	Overall (*N* = 70)	*P* value
DV-QOL Physical	70				.26*
Median (IQR)		4.5 (3.3–5.3)	3.7 (2.3–5.7)	3.8 (3.0–5.4)	
Range		1.7–8.0	0.4–9.2	0.4–9.2	
DV-QOL Concerns	70				.35*
Median (IQR)		7.0 (5.3–8.0)	5.7 (4.0–8.0)	6.0 (4.6–8.0)	
Range		3.3–10.0	1.7–10.0	1.7–10.0	
DV-QOL Emotions	70				.12*
Median (IQR)		5.8 (4.0–6.5)	4.5 (3.0–6.1)	5.0 (3.5–6.5)	
Range		1.9–8.5	0.0–9.0	0.0–9.0	
DV-QOL Behavior	70				.06*
Median (IQR)		5.6 (4.0–6.4)	4.8 (2.8–6.0)	4.8 (3.2–6.0)	
Range		2.0–7.6	0.0–8.8	0.0–8.8	
DV-QOL Overall	70				.12*
Median (IQR)		5.3 (4.7–5.8)	4.7 (3.3–5.9)	4.8 (3.6–5.9)	
Range		2.4–7.3	0.7–8.2	0.7–8.2	

N is the number of nonmissing values.

*DV-QOL*, Diverticulitis Quality of Life Instrument; *IQR*, interquartile range; *SD*, standard deviation.

* Wilcoxon.
