# Supplementary material for: The use of patient-reported outcome measures in recurrent diverticulitis
Source: Surgery. Author manuscript; Available in PMC 2026 Jun 8. (PMC13245719; doi:10.1016/j.surg.2025.109492)
Supplement: 1 [file NIHMS2176722-supplement-1.pdf]

ID# _______ Date __________

DIVERTICULITIS QUALITY OF LIFE QUESTIONNAIRE (DV-QOL)

Instructions

This questionnaire asks you about your quality of life, or "how you feel," with diverticulitis. There are questions about how diverticulitis may affect you physically, emotionally, and socially. Please answer all of the questions the best you can. If you are uncertain of an answer, then write down your best guess. There are no right or wrong answers to these questions. In answering the questions, think about your experiences with diverticulitis during the **past 2 weeks only**.

Thank you!

DV-QOL v1.0 © CS-CORE

Some people say they have episodes of belly pain *caused by their diverticulitis* – that is, pain in the red area shown in the picture below. These episodes are times when belly pain comes on fast or becomes worse than usual. Below are questions about these episodes. In answering these questions, think about the past 2 weeks only.

For these questions, "belly" means the area in red, below.

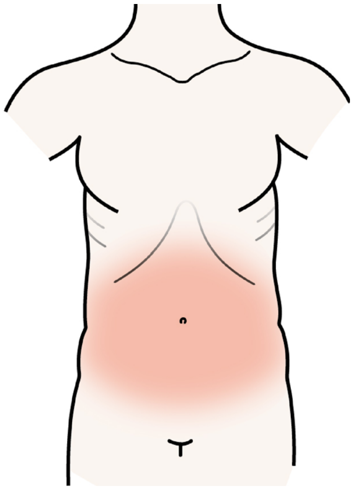

These questions ask about belly symptoms, <u>in general</u>, during the past 2 weeks. In answering these questions, think about any belly symptoms *<u>caused by your diverticulitis</u>* that you have experienced.

Below is a list of symptoms. Read each one and mark the box that best describes <u>how many days</u> you felt that way <u>in the past 2 weeks</u>. Mark only one box for each question and do not skip any questions. It is okay to make your best guess if you are not sure of the exact number of days you had a symptom. If you did not feel that way in the past 2 weeks, mark "no days".

	In the past 2 weeks, how many days…	No Days	1 Day Only	2-5 Days	6-9 Days	10-13 Days	Every Day
S1	Did you <u>feel</u> bloated – that is, <u>feel</u> fullness in the belly?						
S3	Did you have loose or watery stools?						
S6	Did you feel like you needed to pass a bowel movement but could not get it all out?						
S7	Did you have nausea – that is, feel you were about to vomit?						
S8	Did you have belly pain?						

→ **If you answered "1 day only" or more for belly pain (question S8), then answer question S9, below. Otherwise skip to next section [<u>may be automated in online registry or e-diary</u>]**

S9. Below is a picture showing the front of the body. The belly is divided into 9 areas, numbered "1" through "9." Select the areas where you felt your diverticulitis belly pain at least once in the past 2 weeks. You may select <u>more than one area</u> if you had pain in more than one area.

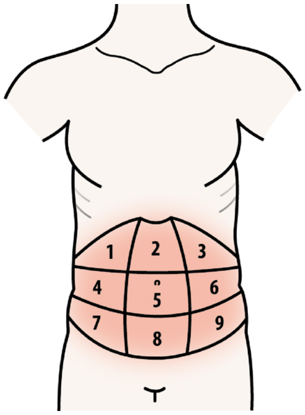

Area 1 [ ]	Area 2 [ ]	Area 3 [ ]
Area 4 [ ]	Area 5 [ ]	Area 6 [ ]
Area 7 [ ]	Area 8 [ ]	Area 9 [ ]

Below is a list of possible concerns about diverticulitis. Please read each statement and mark the box that best describes how much you felt that concern, if at all, in the past 2 weeks. Mark only one box for each statement and do not skip any questions. If you never felt that way in the past 2 weeks, mark "Not at All Concerned."

	In the past 2 weeks, how concerned were you that...	Not at All Concerned	A Little Bit Concerned	Moderately Concerned	Quite a Bit Concerned	Extremely Concerned
C1	Your diverticulitis might flare up or get worse at any time					
C3	Something serious might be wrong with your body because of your diverticulitis					
C6	Your diverticulitis was causing damage within your body					

Below is a list of statements about how diverticulitis might make you feel socially or emotionally. Please read each one and mark the box that best describes how frequently you felt that way in the past 2 weeks. Mark only one box for each statement and do not skip any questions. If you never felt that way in the past 2 weeks, mark "None of the Time."

	In the past 2 weeks ...	None of the Time	Rarely	Sometimes	A Lot of the Time	All of the Time
F2	I felt like others were looking down at me because of my diverticulitis symptoms					
F4	I felt frustrated because of my diverticulitis					
F6	I felt anxious because of my diverticulitis					
F7	I felt irritated because of my diverticulitis					

Below is a list of statements about how diverticulitis might affect what you do. Please read each one carefully and mark the box that best describes how frequently, if ever, you behaved that way in the past 2 weeks. Mark only one box for each statement and do not skip any questions. If you never felt that way in the past 2 weeks, mark "None of the Time."

	In the past 2 weeks ...	None of the Time	Rarely	Sometimes	A Lot of the Time	All of the Time
B2	I ate less food than usual because of my diverticulitis					
B4	I had to wear looser clothing than usual because of my diverticulitis					
B6	I had to avoid social engagements because of my diverticulitis					
B10	I couldn't sleep because of my diverticulitis symptoms					
B11	I had to miss work or other important obligations because of my diverticulitis					

***** END OF QUESTIONNAIRE *****
